# Seuil de positivité de la troponine i cardiaque dans le diagnostic de l’IDM péri-opératoire après chirurgie cardiaque sous circulation extra-corporelle chez l’adulte

**DOI:** 10.11604/pamj.2018.29.40.12900

**Published:** 2018-01-17

**Authors:** Asmâa Biaz, Mohammed Drissi, Aissam El Maataoui, Samira El Machtani Idrissi, Sanae Bouhsain, Abdellah Dami1, Abdellatif Boulahya, Zohra Ouzzif

**Affiliations:** 1 Service de Biochimie, Toxicologie Hôpital Militaire d’Instruction Mohammed V Rabat, Maroc; 2 Service de Chirurgie Cardio-Vasculaire Hôpital Militaire d’Instruction Mohammed V Rabat Maroc; 3 Faculté de Médecine et de Pharmacie de Rabat, Université Mohamed V Souissi Rabat, Maroc; 4 Université Ibn Zohr, Faculté de Médecine et de Pharmacie d’Agadir, Département de Biochimie et de Biologie Moléculaire

**Keywords:** Troponine Ic, chirurgie cardiaque, circulation extra corporelle, infarctus de myocarde, adulte, diagnostic, Troponin lc, cardiac surgery, on-pump cardiac surgery, myocardial infarction, adult, diagnosis

## Abstract

**Introduction:**

L'objectif de cette étude prospective, réalisée sur une année au laboratoire de biochimie de l'HMIMV de Rabat, vise à étudier la cinétique de la Troponine Ic (TnIc) après chirurgie cardiaque sous circulation extracorporelle (CEC) en vue d'établir des valeurs seuils pour le diagnostic d'infarctus de myocarde péri-opératoire (IDMPO).

**Méthodes:**

Nous avons inclus 58 patients opérés pour chirurgie valvulaire ou pontage coronarien sous CEC. Ces patients ont été séparés en 3 groupes selon l'évolution clinique, biologique (TnIc) et électrique durant la période post opératoire précoce. Nous avons dosé et suivi la cinétique de la TnIc par une technique immuno-enzymatique, au moyen du réactif Flex^R^ CTNI avant et après la CEC, à H_0_, H_3_, H_6_, H_12_, H_21_, H_24_ et H_72_ sur l'automate Dimension Xpand plus^R^ de la société Siemens.

**Résultats:**

Les résultats obtenus sont plus élevés qu'en cardiologie, même chez le groupe des patients sans complications cardiaques post opératoires, avec un taux moyen de TnIc environ 2,5 fois supérieur à la valeur seuil en cardiologie. La cinétique de libération est significativement différente entre les 3 groupes (p‹0,05).

**Conclusion:**

La valeur seuil que nous proposons pour confirmer le diagnostic de l'IDMPO est de 13ng/ml obtenue entre H12 et H24. Nous recommandons, de ce fait, un à deux prélèvements vers H12 puis entre H20 et H24.

## Abstract (en)

**Introduction:**

This prospective study was conducted at the Laboratoy of Biochemistry of the HMIMV, Rabat, over a period of 1 year. It aimed to analyse the kinetics of troponin lc (cTnl) after on-pump cardiac surgery in order to establish threshold values for the diagnosis of perioperative myocardial infarction.

**Methods:**

The study included 58 patients who had undergone on-pump valvular surgery or on-pump coronary artery bypass surgery. These patients were separated into 3 groups according to their clinical and electrical evolution as well as their laboratory tests (cTnI) during the immediate post-operative period. We batched and followed the kinetics of cTnl using immuno-enzymatic technique, CTNI Flex^®^ reagent, before and after on-pump cardiac surgery, at H0, H3, H6, H12, H21, H24 and H72 using automatic SIEMENS Dimension Xpand Plus.

**Results:**

The results were higher than in cardiology, even among the group of patients without postoperative cardiac complications, with an average cTnl rate of approximately 2.5 times higher than the threshold value in cardiology. The kinetics of release differed significantly among the 3 groups (p‹ 0.05).

**Conclusion:**

The threshold value that we propose to confirm the diagnosis of perioperative myocardial infarction is 13ng/ml obtained between H12 and H24. We recommend to collect 1-2 samples at H12 and then between H20 and H24.

## Introduction

L'European Society of Cardiology (ESC) et l'American Heart Association (AHA) ont établi des critères pour définir l'infarctus du myocarde (IDM), basés sur la clinique et l'ECG. Ces critères ne sont pas toujours pathognomoniques, d'où le besoin d'un autre outil plus spécifique. L'isoforme cardiaque de la troponine (TnIc et TnTc) constitue un marqueur biochimique idéal et prometteur dans le diagnostic des lésions myocardiques ischémiques. Son dosage semble offrir une cardio-spécificité et une sensibilité accrues, comme cela a été souligné par de nombreuses études. Néanmoins, l'élévation de la troponine cardiaque dans la circulation générale, après chirurgie cardiaque, devrait faire la part entre le traumatisme lié aux lésions cardiaques chirurgicales et une nécrose myocardique postopératoire. L'établissement d'une valeur seuil, permettant d'affirmer la nécrose myocardique après chirurgie reste donc un challenge. Le diagnostic des complications cardiaques en chirurgie cardiovasculaire, et notamment l'infarctus du myocarde péri-opératoire (IDMPO), reste difficile à porter après les procédures de chirurgie sous circulation extra-corporelle (CEC). Ce travail vise à étudier la cinétique de libération de la TnIc au cours et après chirurgie cardiaque à différents moments en vue d'établir une valeur seuil qui permettrait de porter le diagnostic de l'infarctus de myocarde péri-opératoire.

## Méthodes

**Figure 1:**
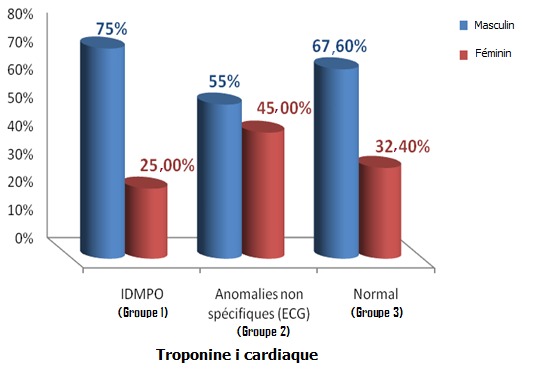
Distribution des trois groupes 1,2 et 3 selon le sexe

Il s'agit d'une étude prospective réalisée à l'HMIMV de Rabat, au laboratoire de biochimie et en étroite collaboration avec l'équipe du service de CCV de la même enceinte. Les patients inclus ont été opérés d'une chirurgie coronaire ou valvulaire sous CEC. Sur 90 patients opérés, seuls 58 ont été inclus dans l'étude, dont 33 remplacements valvulaires et 25 pontages coronariens. Les critères d'exclusion sont l'IDM en préopératoire immédiat, les patients décédés au cours de l'acte chirurgical ou dans les 48H, ceux ayant un taux élevé de TnIc sans anomalies électriques en rapport avec une souffrance myocardique globale et des patients dont le total des prélèvements sanguins n'était pas atteint. Les 58 patients ont été séparés en 3 groupes selon l'évolution clinique, biologique (valeurs de TnIc) et les données électriques durant la période post opératoire précoce: le groupe 1(n = 4) comprend les cas d'IDM péri opératoire, d'âge moyen de 55,25 ans et présentant des troubles de la cinétique segmentaire à l'échocardiographie; le groupe 2 (n = 20, âge moyen de 54,74 ans) présente des signes non spécifiques à l'ECG, avec une sortie de CEC sous catécholamines; le groupe 3(n = 34, âge moyen de 50,70 ans) n'avait aucune anomalie ni trouble à l'ECG. Les autres caractéristiques des patients de la présente étude sont résumées dans le (Tableau 1, Figure 1). Les prélèvements sanguins pour dosage de la TnIc ont été réalisés sur héparinate de lithium à des moments bien définis: au moment de l'induction anesthésique (H_0_), puis à H3, H6, H12, H21, H24 et H72 heures. Ils étaient immédiatement centrifugés à 3000 tours/minute pendant 15 minutes, avant d'être congelés et conservés à -80°C, en fractions aliquotes dans des cryotubes jusqu'aux dosages. Les dosages ont été réalisés sur l'automate Dimension -Xpand plus^R^ de la société Dade-Behring par une technique immuno-enzymatique type sandwich, au moyen du réactif Flex^R^ CTNI de la même société. En respectant les recommandations du fournisseur, la valeur normale et le seuil décisionnel de la TnIc pour l'IDM en cardiologie sont respectivement de moins de 0,04 et de 0,6 ng/ml. Concernant l'analyse statistique, les données ont été saisies et traitées par les logiciels Excel 2007 et SPSS10.0 et les résultats ont été exprimés par la moyenne ± écart type pour les variables quantitatives et par pourcentage pour les variables qualitatives.

**Tableau 1:** Caractéristiques de la population étudiée (n=58)

	Groupe 1n=4	Groupe 2n=20	Groupe 3n=34	p
**Age (ans)**	55,25 ± 3,86	54,74 ± 10,69	50,70 ± 6,45	0,277
**Poids (kg)**	70,75 ± 6,65	66,35 ± 14,66	70,32 ± 14,77	0,602
**Taille (m)**	1,68 ± 0,049	1,67 ± 0,090	1,68 ± 0,064	0,822
**IBM (kg/m^²^)**	25,55 ± 2,34	23,27 ± 4,71	24,82 ± 4,35	0,393
**Durée de clampage aortique (min)**	85,70 ± 25,24	75,25 ±43,07	59,18 ±22,73	**0,015**
**Durée de CEC (min)**	147,75 ± 54,36	126,00 ± 47,37	93,85 ± 28,38	**0,002**

## Résultats

Les résultats des valeurs de la TnIc obtenus sont répertoriés dans le Tableau 2. On note que ces valeurs sont plus élevées pour la chirurgie cardiaque que pour la cardiologie, même chez le groupe des patients normaux sans complications cardiaques post opératoire, chez qui le taux moyen de TnIc est environ 2,5 fois supérieur à la valeur seuil en cardiologie. La cinétique de libération est significativement différente entre les groupes (p‹0,05). L'étude de l'impact du type d'intervention chirurgicale sur les valeurs de TnIc de la population étudiée, à différents moments de la cinétique du marqueur montre des résultats chiffrés plus élevés en cas de remplacement valvulaire, mais il n'existe pas de différence statistiquement significative (p = 0,291). Cette différence demeure non significative même après ajustement de la durée de clampage aortique (p = 0,232) ou encore de la durée de CEC ( = 0,391). En revanche, nous avons objectivé une différence statistiquement significative entre les valeurs moyennes de la TnIc selon le type d'intervention dans le groupe 3 seul, avec p = 0,004. Les taux de TnIc sont retrouvés significativement plus élevés chez les patients ayant subi un remplacement valvulaire que chez ceux opérés pour pontage coronaire. Dans les groupes 1 et 2, la différence entre les moyennes des valeurs de TnIc n'est pas significative en égard du type d'intervention (respectivement p = 0,829 et p = 0,398). Il est également important d'évaluer l'impact de la défibrillation électrique, souvent nécessaire après le déclampage de l'aorte, réalisé de manière spontanée ou par choc électrique interne. Cette évaluation a été réalisée dans le groupe normal, en vue d'éliminer l'interférence possible des facteurs sources de variation du taux de TnIc. Les résultats montrent qu'il n'existe pas de différence significative (p = 0,253) entre les valeurs moyennes de la cinétique de TnIc pour tous les moments d'intervention chirurgicale quel que soit le type de défibrillation électrique. Le seuil de normalité de la TnIc chez les patients du groupe 3 permettant d'exclure l'IDM péri opératoire chez les patients opérés pour chirurgie cardiaque dans la population étudiée est de 3,70 ± 1,87 /ml. Il est défini comme étant la valeur maximale moyenne de TnIc obtenue au cours de l'étude de sa cinétique de libération dans le groupe normal. Dans notre cas cette valeur a été retrouvée à la 12^ème^ H en post opératoire. Afin d'établir la valeur seuil de positivité de la TnIc pour le diagnostic d'IDMPO, une courbe ROC a été établie et a permis de proposer la valeur de 13,22 ng/ml, obtenue à la 24^ème^ H post opératoire dans le groupe 1, comme seuil de positivité. Celui-ci présente une sensibilité et une spécificité de 100%, avec une aire sous la courbe égale à 1 et une valeur de p = 0,001. Comparativement à la valeur seuil utilisée en cardiologie, ce seuil est donc environ 22 fois plus élevé.

**Tableau 2:** Valeurs moyennes de la TnIc à différents moments de la cinétique de sa libération dans les trois groupes

Valeurs moyennes de TnIc (ng/ml)	IDMPO(groupe 1)	Anomalies non spécifiques à l’ECG(groupe 2)	Normal(groupe 3)	p
**TnIc H_0_**	0,25±0,43	0,15±0,23	0,1±0,21	< 0,05
**TnIc H_3_**	5,33±6,5	3,36±3,22	1,38±1,24	< 0,05
**TnIc H_6_**	10,01±11,58	9,49±5,45	2,30±1,44	< 0,05
**TnIc H_12_**	13,71±8,85	13,21±4,90	**3,70±1,87**	< 0,05
**TnIc H_21_**	18,40±6,56	**14,70±5,17**	2,92±1,81	< 0,05
**TnIc H_24_**	**27,41±7,40**	8,98±5,76	2,18±1,63	< 0,05
**TnIc H_72_**	22,65±8,89	3,98±2,98	1,03±1,26	< 0,05

## Discussion

Dans la présente étude, le groupe 1 comprend 4 patients, dont 3 opérés pour pontage coronaire et un pour remplacement valvulaire. Le nombre de patients dans les groupes 2 et 3 est respectivement de 20 et de 34. Cette répartition est à peu près comparable à celle retrouvée dans d'autres travaux, notamment celui d'Alyanakian et al en 1998 [1], portant sur un effectif proche du notre (41 patients). Des résultats différents ont été objectivés par d'autres études [1, 2], celles de Cauliez et al en 2004 et de Madi-Jebara et al en 2006. La survenue d'un infarctus, défini par l'apparition de nouvelles ondes Q sur au moins deux dérivations contigües à l'ECG, est très rare en post opératoire de chirurgie cardiaque et de diagnostic très difficile. En effet, les signes cliniques sont absents et l'ECG montre rarement de nouvelles ondes Q dans un territoire. L'association avec d'autres signes évocateurs reste fondamentale. Le plus souvent l'ECG montre des troubles de repolarisation qui manquent de spécificité et de sensibilité [3]. L'échographie apporte d'autres orientations diagnostiques. Toutes ces limites expliquent qu'en pratique clinique les marqueurs biochimiques restent largement utilisés. La plupart des études se sont intéressées aux concentrations sériques de la TnIc. Dans le présent travail, le profil de libération de TnIc diffère significativement entre les trois groupes. Dans le groupe 3, tous les patients ont eu un profil de libération similaire caractérisé par une augmentation des concentrations de TnIc jusqu'à l'obtention du pic à H_12_ (3,70 ± 1,87ng/ml). Ces concentrations restent significativement plus basses comparativement au groupe 1 et 2. Dans le groupe 2, le pic de libération a été obtenu à H21 atteignant une valeur de TnIc de 14,70 ± 5,17 ng/ml, soit à peu près 5 fois la valeur dans le groupe normal. En revanche, la concentration de ce marqueur au pic était encore plus élevée dans le groupe 1, avoisinant un chiffre de 27,41 ± 7,14 ng/ml (près de 7 fois la valeur dans le groupe 3) à H_24_ et se maintenant à ce taux jusqu'à H_48_. Des résultats comparables ou peu différents ont été objectivés par d'autres études, notamment celles d'Alyanakian et al en 1998 et Cauliez et al en 2004, soulignant la cinétique retardée de libération de la TnIc et/ou des taux plus élevés de ce marqueur dans les deux groupes 1 et 2 comparativement au groupe 3. Ainsi, une augmentation de la concentration de TnIc entre H_12_ et H_24_ est suggestive de dégâts myocardiques. En effet, en absence de souffrance myocardique sévère, les concentrations plasmatiques de troponine (I ou T) restent basses, avec en général un pic précoce, entre H_6_ et H_10_, puis elles redescendent lentement pour devenir indétectables au-delà du 5^ème^ et du 7^ème^ jour [4]. A l'inverse, en cas de souffrance myocardique sévère, les concentrations de TnIc vont progressivement augmenter pour atteindre un pic entre H_12_ et H_24_, suivi d'un plateau et d'une descente plus lente que précédemment [1, 5]. Cette cinétique est assez comparable à celle observée au cours des infarctus en cardiologie. L'analyse de la littérature et la connaissance de la cinétique, en cas de souffrance myocardique ischémique, laissent à penser qu'à partir de H_10_-H_12_, les dosages permettent de mieux discriminer les patients, avec ou sans nécrose péri opératoire. Ceci est particulièrement vrai en chirurgie cardiaque où les temps précoces sont plus difficiles à interpréter, car très influencés par le traumatisme chirurgical. En pratique, selon nos résultats, il convient de recommander un ou deux prélèvements vers H_12_ puis entre H_20_ et H_24_. Dans notre étude, la valeur seuil permettant le diagnostic de l'IDMPO a été déterminée à l'aide de courbes ROC faites entre le groupe normal et le groupe de patients ayant développé un IDMPO. Pour une sensibilité et une spécificité de 100%, la valeur seuil obtenue est de 13,22 ng/ml avec une aire sous la courbe de 1. Nos résultats concordent avec ceux obtenus dans d'autres études [6]. Celles-ci ont montré qu'en chirurgie cardiaque avec CEC, les seuils de positivité se situent entre 12 et 15 ng/ml. En dessous de ce seuil, il n'existe pas de complications cardiaques et l'élévation de la troponine n'est due qu'au dommage myocardique consécutif à la CEC, la cannulation et la cardioplégie [7]. En revanche, dans l'étude de Cauliez et al [2], la valeur seuil de TnIc de 7ng/ml (spécificité de 90,5%) a été retrouvée chez les patients ayant subi un pontage coronaire. En utilisant la même technique de dosage pour la TnIc, les valeurs rapportées dans la littérature sont comprises entre 3,9 et 15ng/ml [1, 8, 9]. Plusieurs facteurs expliquent cette disparité de la valeur du seuil de positivité pour l'IDMPO soulignant la nécessité pour chaque laboratoire d'établir sa propre valeur seuil en chirurgie cardiaque [4]. Parmi ces facteurs il y a le relargage systématique des marqueurs biochimiques par la chirurgie, l'ischémie systématique pendant le clampage aortique. L'incidence est variable selon l'examen de référence et la valeur seuil est variable en fonction de la méthode statistique utilisée. La cinétique peut aider au diagnostic, avec la présence d'un pic tardif vers la 20^ème^ heure en cas de souffrance ischémique significative. Malgré cette difficulté à établir des valeurs seuils très précises, toutes les études retrouvent bien une association entre l'élévation des troponines après chirurgie cardiaque et la survenue de complications en postopératoire [1, 8, 10].

## Conclusion

Les procédures de chirurgie cardiaque sous CEC se sont développées, permettant une prévention des complications cérébrales. Néanmoins, les complications cardiaques demeurent fréquentes et difficiles à diagnostiquer en période postopératoire, sachant que l'échographie et l'ECG sont de mauvaise spécificité. A l'heure actuelle le dosage des troponines cardiaques et notamment TnIc semble le plus adapté du fait de sa grande spécificité et sa meilleure sensibilité. La libération de ce marqueur, en l'absence de toute complication cardiaque post opératoire est d'autant plus importante que le clampage aortique est long et que la durée de CEC est prolongée. Elle dépend aussi du type d'intervention, de la qualité de la protection myocardique et du chirurgien qui opère. La valeur seuil que nous proposons pour confirmer le diagnostic de l'IDMPO est de 13ng/ml obtenue entre H_12_ et H_24_. Nous recommandons, de ce fait, un à deux prélèvements vers H_12_ puis entre H_20_ et H_24_. Toutefois, ces résultats doivent être confirmés par une étude menée sur un grand effectif de patients.

### Etat des connaissances actuelle sur le sujet

- L'isoforme cardiaque de la troponine (TnIc et TnTc) constitue un marqueur biochimique idéal et prometteur dans le diagnostic des lésions myocardiques ischémiques.

### Contribution de notre étude à la connaissance

- Nous proposons une valeur seuil pour le diagnostic de l'infarctus du myocarde en péri-opératoire;
- Nous donnons des recommandations par rapport aux heures de prélèvement.

## Conflits d’intérêts

Les auteurs ne déclarent aucun conflit d'intérêts.

## References

[1] Alyanakian MA, Dehoux M, Chatel D, Seguret C, Desmonts JM, Durand G, Philip I (1998). Cardiac troponine I in diagnosis of perioperative myocardial infarction after cardiac surgery. J Cardiothorac Vasc Anesth..

[2] Cauliez B, Redonnet M, Darras S, Blanchet D, Ménard JF, Bessou JP, Lavoinne A (2004). Troponine Ic et CK-MB masse en chirurgie cardiaque sous circulation extracorporelle. Ann Biol Clin..

[3] van Vlies B, van Royen EA, Visser CA, Meyne NG, van Buul MM, Peters RJ, Dunning AJ (1990). Frequency of myocardial indium-111 antimyosin uptake after uncomplicated coronary artery bypass grafting. Am J Cardiol..

[4] Guillaume L, Philippe M (2004). In troponines et souffrance myocardique.

[5] Fellahi JL, Leger P (1999). Pericardial cardiac troponin I release after coronary artery bypass grafting. Anesth Anal..

[6] Vermes E, Mesguich M, Houel R, Soustelle C, Le Besnerais P, Hillion ML, Loisance D (2000). Cardiac troponin I release after open heart surgery: a marker of myocardial protection. Ann Thorac Surg..

[7] Lasocki S, Provenchère S, Bénessiano J, Vicaut E, Lecharny JB, Desmonts JM, Dehoux M, Philip I (2002). Cardiac troponin I is an indedeendent predictor of in hospital death after adult cardiac surgery. Anesthesiology..

[8] Carrier M, Pellerin M, Perrault LP, Solymoss BC, Pelletier LC (2000). Troponin levels in patients with myocardial infarction after coronary artery bypass grafting. Ann Thorac Surg..

[9] Gensini GF, Fusi C, Conti AA, Calamai GC, Montesi GF, Galanti G, Noferi D, Carbonetto F, Palmarini MF, Abbate R, Vaccari M (1998). Cardiac troponin I and Q-ware perioperative myocardial infarction after coronary artery bypass surgery. Crit Care Med..

[10] Benoit MO, Paris M, Silleran J, Fiemeyer A, Moatti N (2001). Cardiac troponin I: its contribution to the diagnosis of perioperative myocardial infarction and various complications of cardiac surgery. Crit Care Med..

